# High-Throughput Quantification and Characterization of Dual Payload mRNA/LNP Cargo via Deformulating Size Exclusion and Ion Pairing Reversed Phase Assays

**DOI:** 10.1021/acs.analchem.4c06296

**Published:** 2025-01-30

**Authors:** Mateusz Imiołek, Razvan Cojocaru, Szabolcs Fekete, Jon Le Huray, Matthew Lauber

**Affiliations:** † Waters Corporation, Rue Michel Servet 1 Geneva, 1211, Switzerland; ‡ Acuitas Therapeutics, 6190 Agronomy Rd. Suite 405, Vancouver, British Columbia V6T 1Z3, Canada; § Waters Corporation, 34 Maple St., Milford, Massachusetts 01757, United States

## Abstract

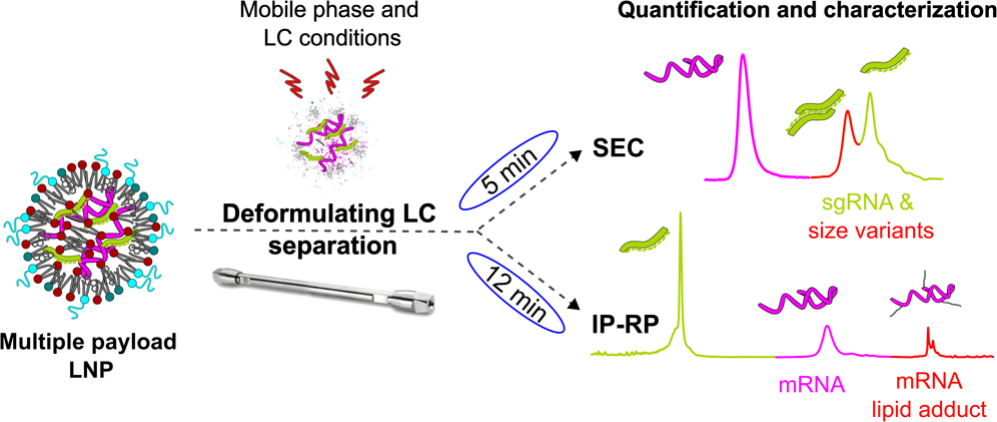

Therapeutic drugs and multivalent vaccines based on the delivery of mRNA via lipid nanoparticle (LNP) technologies are expected to dominate the biopharmaceutical industry landscape in the coming years. Many of these innovative therapies include several nucleic acid components (e.g., nuclease mRNA and guide RNA) posing unique analytical challenges when monitoring the quantity and quality of each individual payload substance in the formulated LNP. Current methods were optimized for single payload analysis and often lack resolving power needed to investigate nucleic acid mixtures. Ion pairing reversed phase (IP-RP) and size exclusion chromatography (SEC) are increasingly being used to characterize nucleic acids. Here, we studied their application for payload quantification in formulated LNP drug-like products. Using a detergent to disrupt the LNPs, the liberated payloads can be separated on an octadecyl RP column using a fast gradient. Reproducible results were obtained as lipids, and surfactants were efficiently eluted using a high organic solvent wash protocol. Alternatively, we also established an online SEC disruption analysis of the mRNA/LNPs wherein an alcohol and detergent containing a mobile phase was applied. Such conditions universally deformulated all tested LNP samples, indicating that a 5 min-long SEC separation can be used as a high-throughput platform method. In both approaches, the measurements facilitate a multiattribute analysis. Apart from quantitation, the characterization of specific impurities is achieved: IP-RP reveals mRNA-lipid adducts, while SEC informs on size variants, which in turn reduces a laboratory’s analytical workload. These easy-to-adopt LC-based assays are expected to fortify the analytical toolbox for emerging gene therapeutics.

## Introduction

Following the remarkable success of COVID-19 vaccines, it is the next generation of nucleic acid-based medicines that holds great promise for addressing currently intractable genetic diseases and cancers. However, addressing complex clinical needs means development of increasingly intricate drug modalities, which necessitates tackling previously unmet analytical challenges.^1^ For example, extending the functionality of these drug products toward genome editing requires simultaneous delivery of various agents, an mRNA encoding for CRISPR-Cas nuclease and an associated guide RNA.^2^ Derisking the development of such mRNA drugs involves multidisciplinary efforts with an important role for analytical characterization of the nanoparticle delivery systems and their payloads.^3^ At present, the available analytical toolbox does not adequately address certain needs, such as monitoring for mixed payload quantities and ratios, which is important to control ribonucleoprotein complex assembly and gene editing efficiency.^4^ Therefore, it is critical to establish new analytical assays that can yield detailed information on LNPs with multiple nucleic acid payloads in a high throughput and straightforward manner.^5^ While measurement of the drug substance alone is relatively straightforward (e.g., via UV absorption or PCR-based methods), the same measurement cannot be as simply performed on the drug product. LNPs, despite being non-UV chromophores at a wavelength of 260 nm, give rise to significant signal due to scattering of the incident light.^6^ The effect is strongly sample dependent (affected by size, polydispersity, lipid composition etc.), which limits the utility of introducing complex corrective factors.^7^ In addition, PCR-based assays cannot be directly performed on encapsulated nucleic acids. The most commonly used and accepted quantification approach is a standard RiboGreen assay (which measures RNA induced fluorescence of free RNA in intact sample and total RNA in a detergent disrupted sample).^8,9^ This method, although it can be realized in a high throughput manner, is not without drawbacks, as it can be quite variable and does not have the specificity to distinguish between different RNA payloads. A nondestructive size exclusion chromatography separation coupled to multiangle light scattering, UV and differential refractive index detectors (SEC-MALS-UV-dRI) method provides size-based payload analysis,^6^ but its use is limited to rapid analyses and operationally more difficult due to the need of empty LNP reference to account for the light scattering contribution in the UV signals. Neither can differentiate between multiple encapsulated cargos, only yielding information about the total (or combined) content. As a general strategy, the payload can be isolated from the LNPs (and separated with a generic nucleic acid method), but this often involves convoluted sample preparation protocols that may introduce artifacts to the analysis as well as prohibitively decrease the throughput.^10^ In addition, the RNA extraction protocol needs to be highly efficient to be suitable for application to quantitative assay. Carefully optimized multiplexed assays based on sequence recognition are another option, but there is an upfront labor and validation cost.^11^ Apart from such laborious methods, one can attempt assays based on separation of the analytes such as capillary gel electrophoresis (CGE)^12^ or various liquid chromatography techniques. However, such methods require further validation and testing against different LNP formulations to extend and validate their use. Chromatographic separations, typically performed in a high-throughput fashion without assay-specific training and applicability to a general class of analytes, represent a potentially attractive solution to the challenge of multiple payload quantification. Among several available options, size exclusion chromatography (SEC) and ion pairing reversed phase chromatography (IP-RP) are increasingly useful for the characterization of emerging new modalities (AAVs, nucleic acids, mRNA/LNPs etc.).^13,14^ In their current form, they are effective tools for enabling fast and efficient quantification of free nucleic acid drug substance,^15,16^ in contrast to anion exchange chromatography, which can suffer from large carryover between injections.^17^ Both methods can be used according to the available analytical capacity and the specific character of the samples. While IP-RP typically offers higher resolution for tailored methods, especially for smaller nucleic acids,^18^ SEC with its size based separation mechanism on modern low adsorption columns has the advantage of requiring little to no method development.^19^

Release of the encapsulated nucleic acid requires disruption of the stable LNP particle, effectively leading to denaturing chromatography. In general, reversed-phase liquid chromatography (RPLC) under disruptive conditions can be used to measure the total RNA content of the LNP samples. Without ion-pairing (nonretentive conditions), there is no/little separation between different payloads.^6^ On the other hand, there is a chance to differentially retain and elute these components by using ion pairing agents (alkylamines) and optimized gradient elution methods. However, the brief application of high temperature and low organic cosolvent initial conditions of an IP-RP method is not sufficient to achieve complete disruption of the LNPs. Even harsher conditions are needed to improve the efficiency of the process. Detergent-based disruption may be called for, but conditions need to be carefully investigated to ensure results are obtained that are in agreement to those determined by orthogonal methods.^20^ This is not trivial since surfactants loaded onto a column are expected to cause poor reproducibility, interference peaks, and short column lifetime. Being nondenaturing in their nature (i.e., lower temperature and not comprising organic solvent), one would predict that SEC separations would require even harsher conditions to ensure disruption of the LNPs. In general, analysis of intact LNPs is complicated by the strength of secondary interactions of particle components and has previously required custom-made solutions that only allows fractionation of certain samples.^21^ Until recently, hardware limitations restricted the use of analytical SEC for nucleic acids, but the growing body of literature suggests that using modern (chemically inert) columns can lead to efficient analyses of even large nucleic acids.^18,19,22^ To the best of our knowledge, successful SEC and IP-RP analyses of deformulated LNPs have not been described, likely due to challenges related to incomplete disruption and low robustness of detergent aided analyses.

To expand the available repertoire of analytical methods for the quantification of LNP payloads, we optimized sample deformulation protocols and IP-RP conditions for reliable quantification of multiple payload LNPs together with any associated lipid adduct impurities. Noting the higher complexity of running deformulating IP-RP, we additionally developed conditions for the complete online disruption of LNPs during SEC separations. Both enable the direct measurement of dual nucleic acid payloads. We propose the use of a deformulating SEC mobile phase consisting of low levels of sodium dodecyl sulfate and isopropanol. This allows direct injections of intact LNP samples into the LC flowpath, where it is dissolved into its individual components. The conditions of both the IP-RP and SEC assays are shown to be universal for all tested formulations and payloads. The methods were verified to be specific, linear, and robust, yielding not only quantification of several payloads but also characterization of their size variants.

## Material and Methods

Chemicals and solvents were used as received, including phosphate buffered saline pouches from Sigma-Aldrich (P3813), isopropanol (IPA) Optima LC-MS grade from Fisher Chemical (10684355), water UHPLC gradient grade from Fisher Chemical (11357090), sodium dodecyl sulfate (SDS) from Sigma-Aldrich (L3771), Triton X-100 Surfactant from Sigma-Aldrich (X100), 20× TE Buffer from Thermo Fisher (T11493), nuclease-free water from Thermo Fisher (4387936), Acetonitrile Optima LC-MS grade from Fisher Scientific (A955), Triethylammonium Acetate, from Millipore-Sigma (625718), Dibutylammonium Acetate from TCI Chemicals (A5702), and Triethylamine from Fisher Scientific (O4884500). The LNP samples were either obtained from commercial sources (LNP1: Spikevax COVID-19 vaccine, NDC 80777-279-99, 0.1 mg/mL; LNP2: Comirnaty COVID-19 vaccine, NDC 59267-1055-4, 0.1 mg/mL) or were produced in-house at Acuitas Therapeutics (lipid formulations LNP3, 4, and 5 loaded with 1:1 FLuc mRNA and sgRNA, varying amount of Cas9 mRNA and sgRNA, or siRNA, target concentration 1 mg/mL) according to standard protocols. Empty LNPs of LNP3, 4 and 5, without nucleic acid payloads, were prepared at Acuitas Therapeutics in the same way. Nucleic acids were obtained from GenScript (Cas9 mRNA, eSpCas9-230807A), TriLink (FLuc mRNA, L-7202-BK), Synthego (sgRNA, 00268910), and Thermo Fisher Scientific (siRNA, 4404021). Calibration standards for quantification were used within 24 h of preparation.

### Deformulating SEC Conditions

SEC experiments were performed with an ACQUITY UPLC H-Class Bio QSM System equipped with a TUV detector (Titanium 5 mm flow cell, Waters). The deformulating SEC used diol bonded GTxResolve Premier BEH SEC 450 Å 2.5 μm Columns in 4.6 mm × 150 mm format (Waters). Optimized, universally deformulating SEC conditions use 1× PBS, 20% IPA and 0.2% SDS as the mobile phase flowed at 0.5 mL/min in a 5 min method at 40 °C and 260 nm detection. The aqueous mobile phase was filtered before use with 0.2 μm membranes (PES, Nalgene). In the optimized SEC deformulation protocol, the LNP samples were diluted to 0.1–0.2 mg/mL with nuclease free water (or detergent during the method development) in low adsorption vials (Waters Corporation, Milford, MA) and used without further manipulations. Typically, 50–200 ng of the sample was injected onto the SEC column. We found that using more diluted samples may lead to lower reproducibility of the injections, lowering the precision of the method. For quantification, the average values of triplicate injections were used, and calibration curves were constructed using at least 3 standards diluted to different concentrations.

### IP-RP Disruption Conditions

IP-RP experiments were performed with an ACQUITY Premier System with a QSM, FTN equipped with a PDA eλ detector. Separations were performed using an XBridge Premier Oligonucleotide BEH C18 Column, 130 Å, 2.5 μm, 2.1 mm × 50 mm (Waters). In the optimized IP-RP deformulation protocol, the samples were disrupted via a 2-step process. First, samples were diluted 2-fold in an equal volume of 10% Triton X-100 Surfactant in 2×-TE buffer (20 mM Tris-HCl, 2 mM EDTA, pH 7.5) and incubated at room temperature for 30 min. The resultant mixture was further diluted 5-fold in nuclease-free water prior to IP-RP analysis. For quantification, calibration curves were constructed using 5 standards diluted to different concentrations and injected in duplicate, while sample measurements were averaged from triplicate injections. Typically, 60–600 ng of the sample was injected onto the IP-RP column. Samples were resolved using a 12 min method at 80 °C with 1.0 mL/min flow and 260 nm detection, utilizing 50 mM dibutylammonium acetate (DBAA) and 100 mM triethylammonium acetate (TEAA) as ion pairing reagents with an acetonitrile and isopropanol gradient to wash away the detergent and fully elute the lipids, as described in Table S1. All calibration standards and samples were blank corrected against a representative blank injected at the start of the sequence (Figure S1).

## Results and Discussion

### IP-RP Assay Method Development

IP-RP is a useful tool for high-resolution characterization of nucleic acids in the absence of LNP formulation components. However, it has yet to be applied directly to the analysis of LNP drug products due to the lack of a robust and quantitative deformulation method compatible with the downstream chromatographic analysis. Here, we developed a detergent-based deformulation process and associated IP-RP separation to accurately measure the nucleic acid payloads while washing away LNP lipid components and detergent using a solvent gradient. To aid the deformulation process and achieve maximal denaturing conditions with rapid run times, samples were analyzed on a short, 2.5 μm particle column (2.1 mm × 50 mm) using elevated temperature (80 °C) and high flow rate (1 mL/min). Further, to achieve enhanced resolution between a wide range of oligonucleotides and to resolve RNA-lipid adducts, a combination of triethylammonium acetate and dibutylammonium acetate ion pairing agents were used.^23,24^ With these conditions and an acetonitrile (ACN) gradient, we rapidly resolved RNA species of different sizes: Cas9 mRNA (4521 nt), FLuc mRNA (1929 nt), sgRNA (100 nt), and siRNA (20 bp) (Figure S2). It is worth noting that a standard pore size stationary phase has been purposely employed in this technique. Large mRNA are restricted to adsorptive interactions at the exterior of the sorbent particles while guide RNA or smaller RNA components are able to access intraparticle surfaces.

**Figure 1:**
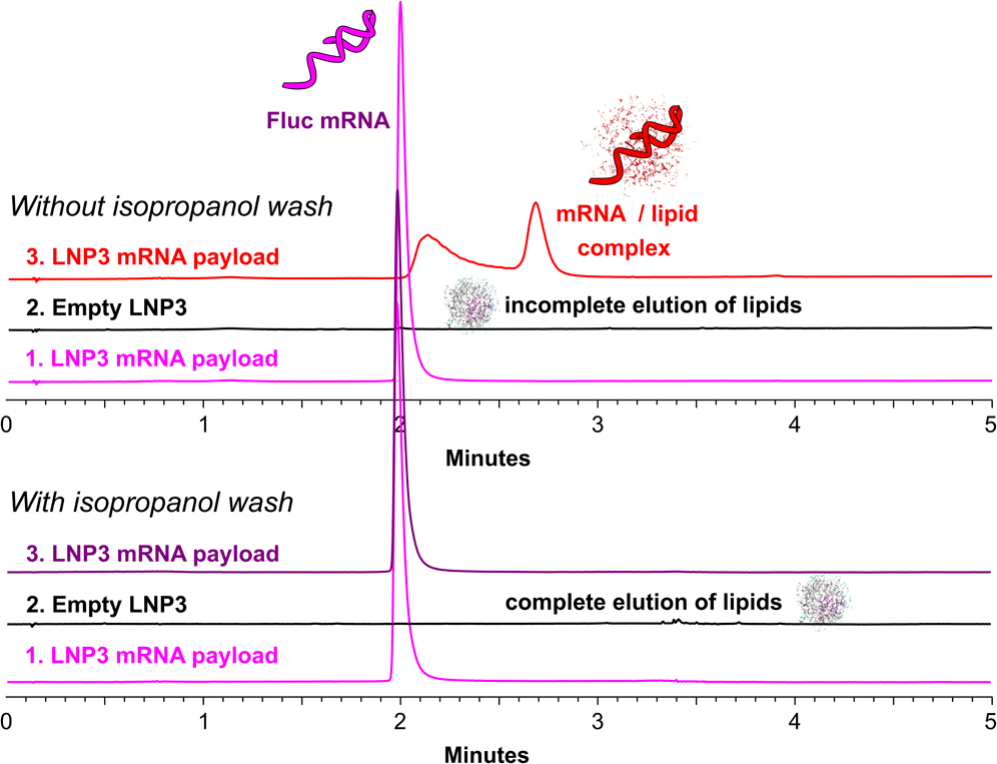
IP-RP method development. Consecutive separations of unencapsulated FLuc mRNA interspersed by an empty LNP injection without (top) and with (bottom traces) an IPA column wash.

To achieve equivalent analysis of LNP encapsulated versus unencapsulated RNA species, we optimized a 2-step deformulation protocol. LNP samples were first disrupted in a buffered solution and high concentration of detergent (sample diluted to 1× TE, 5% Triton X-100 Surfactant) through a 30 min ambient temperature incubation, prior to 5-fold dilution with water for the IP-RP analysis (Final sample composition: 0.1 mg/mL LNP/mRNA, 2 mM Tris-HCl, 0.2 mM EDTA, 1% Triton X-100 Surfactant). These optimized conditions were a result of a wide range of testing and several noted limitations. First, to achieve universal disruption of LNPs, regardless of the ionizable lipid used in the formulation, we wanted to apply the highest possible detergent concentration. However, a titration with Triton X-100 Surfactant showed a significant impact on the RNA peak shape with the increased amounts of the detergent (considerable peak width increase with >1%, Figure S3). To overcome this limitation and maintain maximized disruption capability, we resorted to the 2-step deformulation protocol, effectively disrupting the LNPs with 5% Triton X-100 Surfactant, while injecting the final deformulated sample at diluted 1% detergent concentration. Second, we added an isopropyl alcohol column wash following the RNA separation with the acetonitrile gradient to wash out residual lipids. Applying multigradient elution steps resulted in nonlinear but a highly reproducible baseline, which could be easily corrected with blank subtraction (Figure S1). Without this alcohol regeneration step, we observed high variation of elution times and RNA peak shapes in between injections (Figure 1 - top). The interfering effect of retained lipids was confirmed by the injection of unencapsulated RNA interspersed with empty LNPs. This control experiment led to similarly irreproducible separations, an effect which completely disappeared with the additional IPA wash (Figure 1 - bottom).

Lastly, we observed unexpected highly retained peaks in disrupted LNPs that could not be attributed to lipid-adducts or incomplete disruption, as these peaks were not observed for RNA extracted from the LNP (via offline isopropanol precipitation) but also occurred by spiking in empty LNPs (Figure S4). Therefore, we concluded that these peaks can originate from a strong noncovalent interaction between the ionizable lipid and the mRNA and were able to eliminate this phenomenon by changing the mobile phase pH from 7 to 10 (in which case the mobile phase is used to deprotonate and neutralize the positive charge on the ionizable lipid, Figure S4D).

### IP-RP Results and Discussion

**Figure 2:**
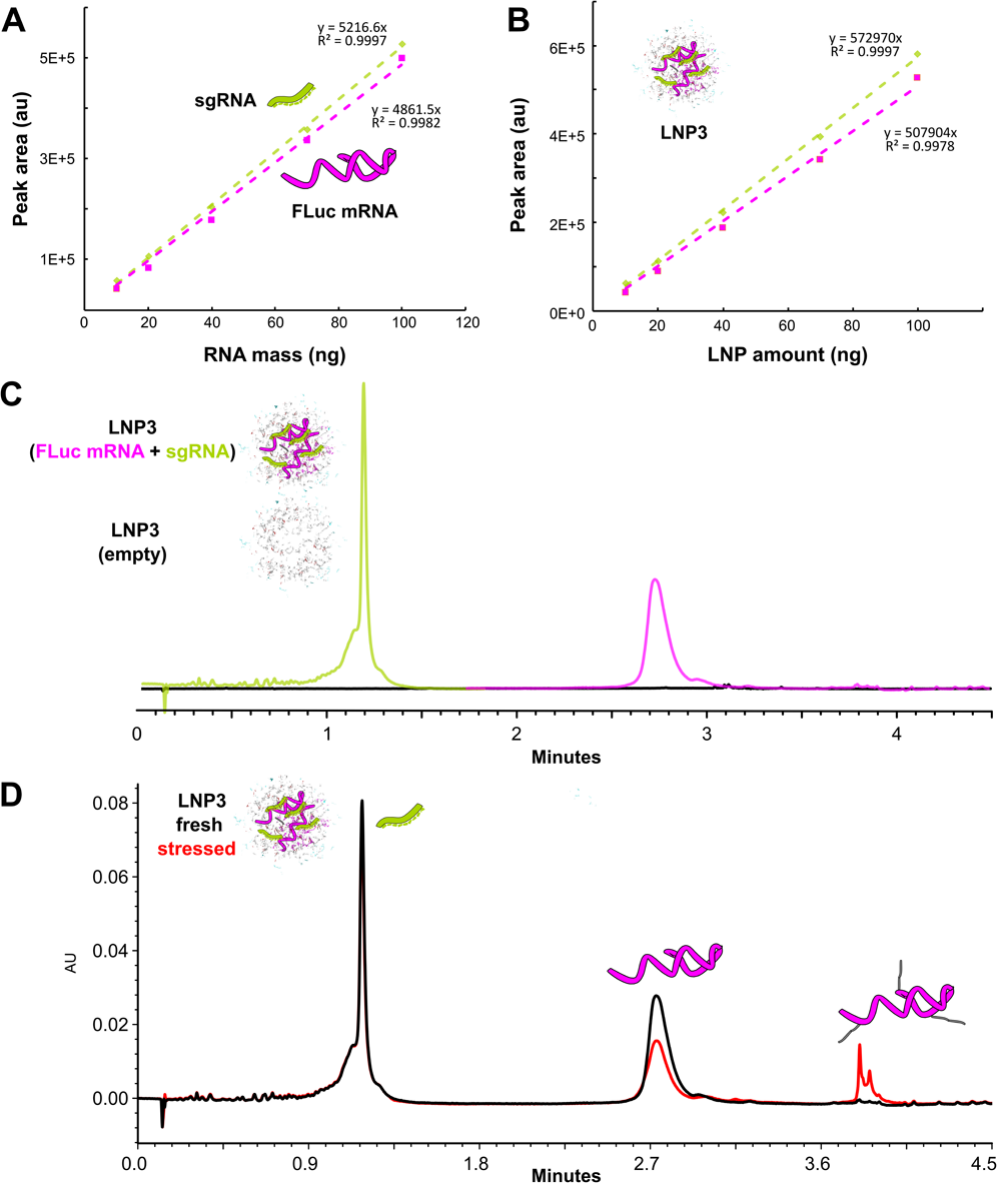
Use of a deformulating IP-RP assay for quantification of dual payload LNPs. A) Calibration curve for reference nucleic acids: sgRNA (green) and FLuc mRNA (fuchsia). B) Linear response of LNP3 loaded with the same RNAs across variable on column mass load. C) Overlay of IP-RP chromatograms showing response of LNP3 loaded with FLuc mRNA and sgRNA or formulated without any payload (empty - black trace). D) Overlay of IP-RP chromatograms for a fresh LNP3 sample (black) and a LNP3 sample stored at room temperature for 1 month (red) showing separation of lipid-adducted mRNA and loss of intact mRNA peak.

The fully optimized method was then assessed and applied to the quantification of several dual and single payload LNP samples. Using a five-point calibration curve ranging from 0.01 mg/mL to 0.1 mg/mL for each component, the method demonstrated good linearity with consistent *R*^2^ > 0.997 over multiple runs (Figure 2A and 2B). We assessed the specificity of the assay by injecting equivalent empty LNPs, observing no detectable signal (Figure 2C). Further, we demonstrated the accuracy of the method through the recovery of sgRNA and FLuc mRNA standards spiked into empty LNPs at concentrations between 0.025 and 0.075 mg/mL (Table S2 and Figure S5). The high precision of the method was assessed through triplicate injections of each preparation in this manuscript, showing RSDs of <4.0%. We measured three formulations containing sgRNA (100 nt) and FLuc mRNA (1929 nt), each containing a different ionizable lipid (LNP3, LNP4, LNP5). The determined ratio and concentration of the formulated samples showed similar results to target values, a previously reported RP method,^6^ and the SEC results discussed in the following sections (Table 1 and Table 2, respectively). We also applied this method to measure four formulations containing sgRNA (100 nucleotides) and Cas9 mRNA (4521 nucleotides) at different ratios. The determined ratio and quantitation were consistent with the formulation targets and SEC results. To show the versatility of the method, we also quantified siRNA (20 bp) filled LNPs, showing high accuracy to the target (Figure S6).

**Table 1:** Results of Quantification of Dual Payload LNP Samples (sgRNA and FLuc mRNA or Cas9 mRNA) Obtained with the Deformulating IP-RP Method and Compared to an Orthogonal Quantification Method for Total RNA (RP Assay)

**Sample**	**Total** [mg/mL]	**RP assay** [mg/mL]	**gRNA** [mg/mL]	**mRNA** [mg/mL]	**Ratio** gRNA/mRNA
**sgRNA + FLuc mRNA**(1:1)					
LNP3	1.03	1.08	0.52	0.51	0.99
LNP4	1.03	1.07	0.52	0.51	0.98
LNP5	1.02	1.02	0.51	0.51	0.99
**sgRNA + Cas9 mRNA (various ratios)**					
LNP3 (1:2)	1.04	0.90	0.35	0.69	0.51
LNP3 (1:1)	1.03	0.86	0.52	0.52	1.00
LNP3 (3:1)	1.07	0.98	0.80	0.27	2.93
LNP3 (5:1)	1.10	1.04	0.91	0.19	4.68

**Table 2:** Results of Quantification of Dual Payload LNP Samples Obtained with the Deformulating SEC Method and Compared to an Orthogonal Quantification Method (RP Assay)

**Sample**	**Total** [mg/mL]	**RP assay** [mg/mL]	**gRNA** [mg/mL]	**mRNA** [mg/mL]	**Ratio** gRNA/mRNA
**sgRNA + FLuc mRNA**(1:1)					
LNP3	1.08	1.08	0.54	0.54	1.01
LNP4	1.06	1.07	0.53	0.54	0.99
LNP5	1.02	1.02	0.50	0.52	0.96
**sgRNA + Cas9 mRNA (various ratios)**					
LNP3 (1:2)	0.96	0.90	0.34	0.62	0.55
LNP3 (1:1)	0.91	0.86	0.47	0.44	1.05
LNP3 (3:1)	0.97	0.98	0.73	0.24	3.04
LNP3 (5:1)	0.99	1.04	0.83	0.16	5.06

Lastly, it was previously identified that ionizable lipid species are a cause of mRNA-lipid adduct formation brought on by oxidative stress and aldehyde intermediates, ultimately leading to loss of protein expression.^23,25,26^ Therefore, it is extremely valuable to be able to distinguish between active mRNA and inactive adducted mRNA. Previously reported adduct quantification methods require >30 min mRNA extraction step prior to chromatographic separation. In contrast, our method can characterize adduct formation in a 12 min run with no need for extraction, while simultaneously quantifying the nucleic acid components (Figure 2D).

Overall, the optimized IP-RP method allows for accurate, extraction-free measurements of RNA in a wide range of LNP products. While tested only on a limited number of samples, the method yielded consistent results across formulations using three different ionizable lipids and pay load sizes ranging from the 20 bp siRNA up to the 4521 nt Cas9 mRNA. Additionally, the method has sufficient resolution to separate mRNA adduct impurities and provide information on the quality of the mRNA in addition to quantification. However, care should be taken when implementing these conditions, because they are beyond the operating conditions of many reversed phase columns. Hydrophobically bonded organosilica, like BEH C18 Particles, is unique in that it can be applied under extreme pH and temperatures. Nevertheless, further optimization of mobile phase pH (e.g., 8.5 vs 10) and column temperature (60 vs 80 °C) may help lengthen the chemical lifetime of the column when used with these newly established assay conditions. An offline deformulation step may need to be adjusted for some LNP formulations.

### SEC Assay Method Development

Recent reports on SEC analyses for mRNA suggest using columns with average pore diameter of 1000 Å, which allows efficient fractionation of common mRNA sizes ranging between 1000–5000 nt.^22,27^ However, in this project, we were mostly concerned about separation power for payloads used in gene editing LNPs: single guide RNA and nuclease mRNA. In this case we intended to maximize the resolution between smaller (∼100 nt) and much larger species (>2000 nt). Our tests revealed that the most appropriate column for this purpose is one with smaller average pore size of 450 Å (larger separation window of 4500 nt and 80 nt RNA and small molecules - Figure S7). Although detailed characterization of most mRNA high molecular weight species (HMWS) is not optimal under these conditions, such a column is still capable of indicating residual aggregate structure of even intermediate size mRNA (shoulder for Cas9 mRNA - Figure S7). Therefore, we decided to develop the assay with a 450 Å, 2.5 μm particle column, which is designed for large cell and gene therapeutics, as recently demonstrated in analyses of viral vectors.^28^ We aimed to use a column that is batch tested for efficiency of nucleic acid separations and employs low adsorption hardware, which is critical for robust analyses of nucleic acids.^29,30^ Such a strategy ensures more facile transfer of the developed method into routine QC laboratories. Additionally, we preferred a column packed with small particles, which improves the efficiency and throughput of the analyses.^19^

**Figure 3:**
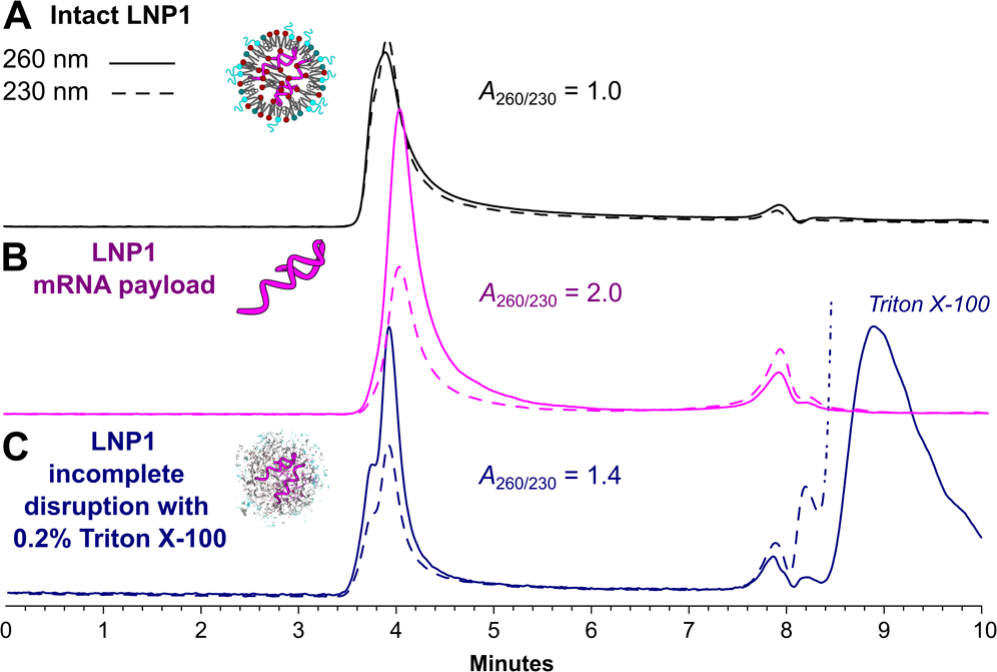
SEC chromatograms showing overlay of two UV signals recorded at 260 nm (continuous line) and 230 nm (dashed line) for A) intact LNP1 sample, B) its extracted mRNA payload, and C) LNP1 samples diluted with 0.2% Triton X-100 Surfactant. Separation was performed under native conditions with 100 ng of the injected sample.

In the first step, we looked at the fractionation of intact LNP particles and their respective payloads. To develop the method, we used commercially available samples filled with single nucleic acids (COVID-19 vaccines LNP1 and LNP2). In order to obtain the payload RNA from LNP1 and LNP2, for which the reference RNA was not readily available, we performed alcohol extraction of the nucleic acid following standard procedures.^23^ SEC separations under native conditions (1× PBS, 25 °C) revealed that both intact LNP1 and its payload elute at similar times (3.91 and 4.03 min, respectively), close to the total exclusion time of the column (Figure 3A and B). This was expected for the 450 Å column, as COVID-19 vaccines LNPs are reported be around 93 nm in diameter^31^ and are filled with long spike protein mRNA (around 5000 nt).

To be able to differentiate between the two species during method development, we decided to record the signal at an additional wavelength of 230 nm and use the fact that pure RNA has a characteristic UV spectrum with a well-defined ratio of the peak (260 nm) and the trough (230 nm) of 1.8–2.2.^32^ Indeed, such an analysis revealed that intact mRNA-LNPs have *A*_260/230_ ratios close to 1, while as predicted, mRNA alone yields values close to 2. A similar conclusion was made upon analysis of LNP2 (Table S3). This result for intact LNPs can be understood in terms of additive scattering component, which is dominant at shorter wavelengths, giving rise to a stronger UV signal at 230 nm.^33^ These data suggest that under native conditions, LNP preserves its structure, which is in line with previous reports using similar column packing material and conditions.^34^ It is nonetheless worth noting that these columns and methods are not optimally designed for the repeat analysis of intact LNPs.

Next, we investigated deformulation protocols that would enable the efficient disassembly of the LNPs and release of the payload substances. As with the IP-RP assay, we started our evaluation with Triton X-100 nonionic Surfactant.^35^ We first evaluated the addition of this detergent at a concentration of 0.2%, observing only partial deformulation at such a level (Figure 3C). While using higher concentrations (up to 2%) increased the deformulation efficiencies (increasing the *A*_260/230_ ratio). Unfortunately, we also observed significantly altered chromatographic profiles (changes in elution time and peak shape as well as intensive residual signal, Figure S8A), suggesting that an alternative approach would be needed. Interestingly, the use of different detergents (SDS, Pluronic F-68 Detergent) as sample diluents led to similar results with incomplete deformulation (*A*_260/230_ < 2) at lower concentrations (0.1–0.5%), as well as poorly reproducible results and peak tailing at higher concentrations (>0.5%) (Figure S8B).

**Figure 4:**
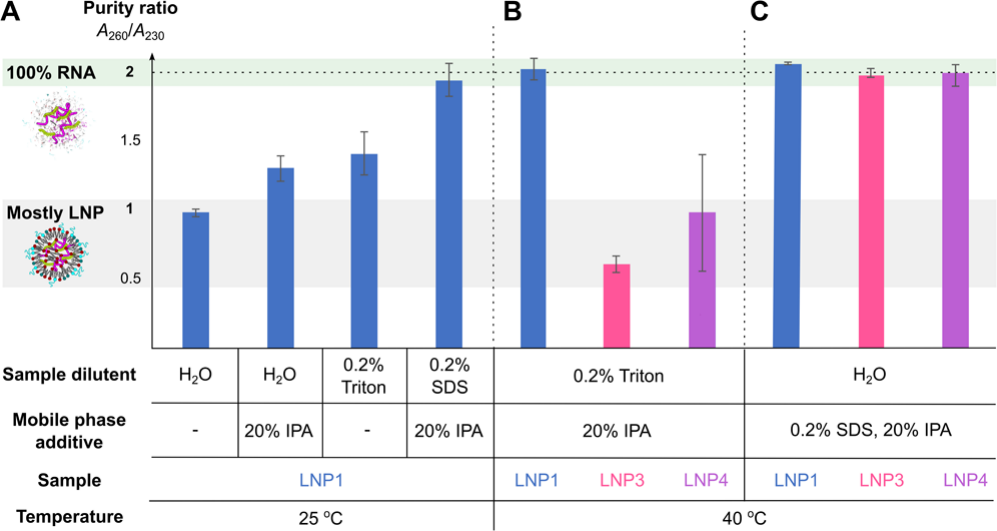
Development of deformulating SEC analysis. A) Comparison of different deformulation conditions by analysis of 260 and 230 nm absorbance ratios (purity ratio). In all cases, the mobile phase based on 1× PBS was used with or without isopropanol (IPA) as the mobile phase additive on a water or detergent diluted sample. B) Comparison of deformulation efficiency between samples with different lipid formulations for Triton X*-*100 Surfactant diluted samples analyzed with IPA as a mobile phase additive. C) Purity ratios under optimized conditions for different intact LNP samples analyzed with 0.2% SDS and 20% IPA mobile phase.

In certain protocols, the LNP sample with detergent is heated to facilitate and accelerate the disruption process. In our hands, such heated samples indeed achieved higher levels of deformulation, but no conditions could be found that produced complete deformulation (via variation of temperature up to 90 °C, time up to 10 min, and amount of detergent up to 0.2%). Importantly, we noticed that integrity of the nucleic acid can be compromised even by short incubation at higher temperatures (Figure S9), confirming previous reports highlighting susceptibility of RNA to degradation, especially for larger species.^27^ As an alternative, we explored the use of organic solvents to aid the deformulation. Disappointingly, dilution with acetonitrile or 2-propanol at low concentrations (up to 20%) indicated only partial deformulation. We decided not to pursue them as sample diluents since at higher concentrations, they restrict the solubility of the nucleic acid component, which would limit the use of the method for precise quantification. Instead, we explored their use as mobile phase additives. Addition of isopropanol to the eluent caused only partial denaturation of LNP1 (isopropanol has been identified as the most disruptive out of tested solvents including MeCN and DMSO); however, when applied to a sample diluted with a detergent (SDS or Triton X-100 Surfactant), the predicted ratio for clean (pure) RNA could be obtained (Figure 4A). Further improvement and reduction of the column pressure was achieved by slight elevation of temperature to 40 °C.

We applied these conditions (sample dilution with 0.2% Triton X-100 Surfactant and 20% isopropanol as a mobile phase additive) to custom, in-house-prepared multiple payload samples loaded with model mRNAs: Cas9 mRNA or FLuc mRNA together with sgRNA (LNP3, 4B). In this case, full deformulation was not achieved, highlighting variability in the deformulation process and a likely dependence on specific formulations. With a limited possibility of increasing detergent or organic solvent content in the sample as well as raising the temperature, we decided to test the addition of surfactants to the mobile phase. To our delight, we observed disruption of all tested LNPs when using 0.2% SDS as the additive (Figure 4C). Finally, we optimized the analysis time with the application of a 0.5 mL/min flow rate, which allowed for a 5 min total separation time (Figure S10, 1.2× column dead-time). In the next step, we verified that such conditions do not introduce artifacts into the analysis. Indeed, we observed the same profile for our series of RNA molecules whether we used native (1× PBS, 25 °C) or deformulating conditions (1× PBS, 20% IPA, 0.2% SDS, 40 °C; Figure S11A).

### SEC Results and Discussion

**Figure 5:**
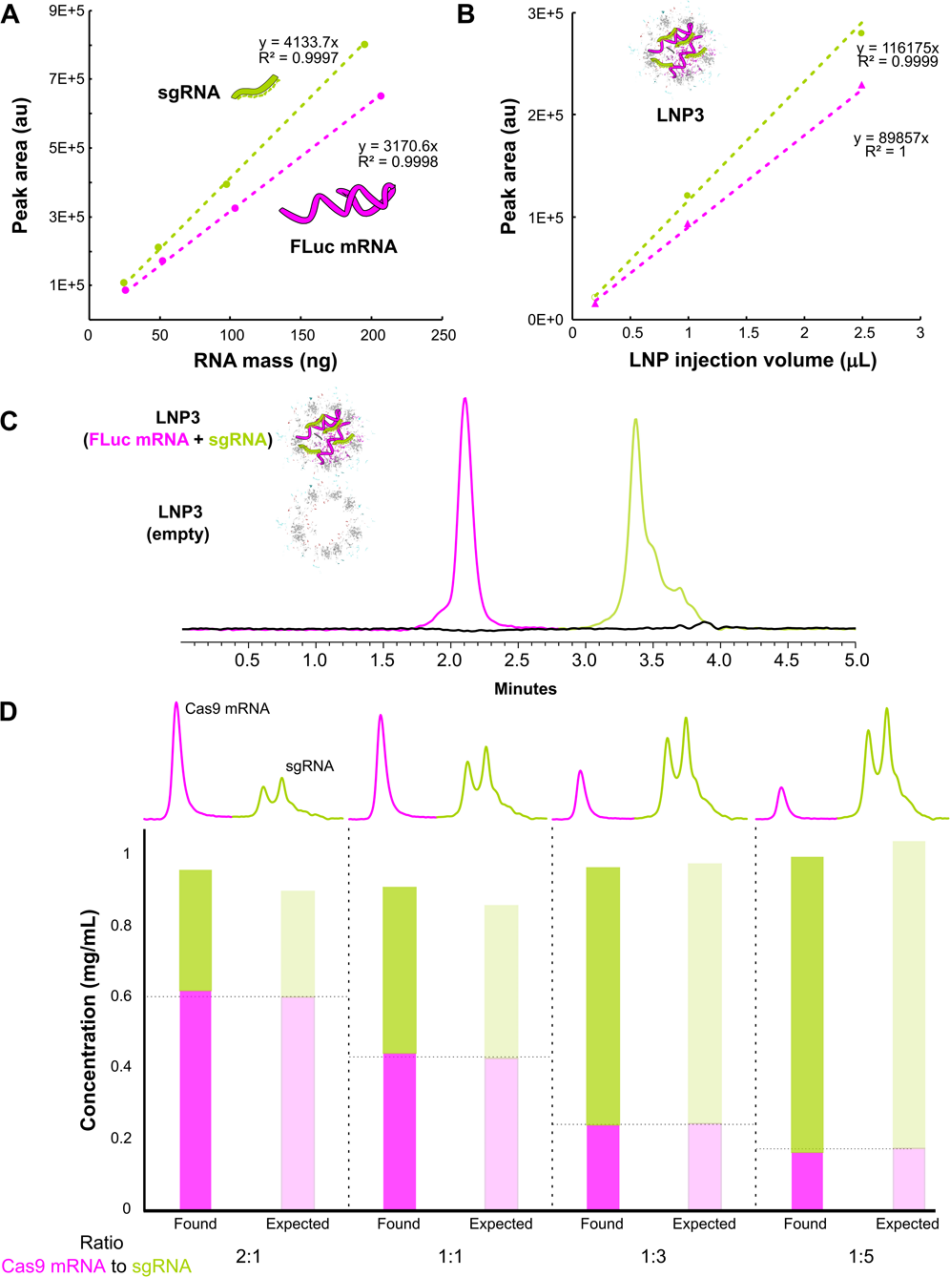
Use of the deformulating SEC assay for quantification of dual payload LNPs. A) Calibration curve for reference nucleic acids: sgRNA (green) and FLuc mRNA (fuchsia). B) Linear response of LNP3 loaded with the same RNAs across variable on column mass load. C) Overlay of SEC chromatograms showing response of LNP3 loaded with FLuc mRNA and sgRNA or formulated without any payload (empty - black trace). D) Quantification of LNP3 filled with a variable amount of Cas9 mRNA and sgRNA compared to the expected value used in formulation together with SEC chromatograms showing separation and relative amount of observed mRNA and sgRNA.

Having established universal conditions for deformulation of LNP nucleic acids, we turned to applying them to the quantification of several dual payload LNP samples containing FLuc mRNA and sgRNA. First, we recorded relevant reference RNA calibration curves (Figure 5A) together with a linearity check for several mass loads of the LNP analytes (Figure 5B). Similarly, we applied this method to LNPs formulated with different ratios of Cas9 mRNA to sgRNA, which allowed us to determine the amount of each payload, revealing that both components were present in the expected ratios at which they were mixed during LNP formulation (Figure 5D). We confirmed the specificity of the assay by injections of empty LNPs, which did not yield appreciable signal (<2% signal in the sgRNA elution time window at a 100% spike level), consistent with the disassembly of UV light scattering LNP particles into non-UV active lipid monomers (Figure 5C and Figure S12A). Finally, standard addition experiments with reference RNAs yielded expected increases in the signal (Figure S12B). We evaluated precision of the method via triplicate injections made on two different columns; RSDs of 1–4% were obtained (Figure S13). The use of a small column format (4.6 × 150 mm) allowed us to reach an LOQ as low as 7 ng (based on FLuc mRNA payload). Moreover, we performed >500 injections of LNP samples on a single SEC column without any noticeable increase in operating pressure or lowered recoveries, indicating the method to be robust.

Additionally, like with the development of the IP-RP assay, we established the total payload concentration of formulated samples using a previously reported RP method.^6^ All results proved to be consistent (Table 2).

Interestingly, during the analysis of Cas9 mRNA filled LNPs, we observed a double peak for sgRNA indicative of the presence of a dimer, confirming the nondenaturing character of the analysis (Figure 5C - chromatograms). Such a species has been commonly observed in literature.^36^ The oligomer was present at the same relative level in all LNPs that were formulated using a specific batch of sgRNA, highlighting the preserving nature of LNP encapsulation. We established that the presence of this aggregate is dependent on the solution conditions of the sgRNA: in low ionic strength solutions, the dimer readily converts into the monomeric species, while in the presence of salts (>15 mM), it becomes markedly more stable. It is also worth noting that we could successfully remove this noncovalent aggregate through heat treatment, confirming previous observations on the dynamic nature of RNA HMWS^34,36^ (Figure S14).

Although the deformulating SEC assay was tested on a limited number of samples, it included representative LNP formulations and samples of FDA approved COVID-19 vaccines. In this way, the tested samples are representative of clinically viable LNPs. To be more confident about the future applicability of this method, we also tested increasing the amount of SDS (up to 1%) and temperature (up to 55 °C), which delivered equivalent results for the tested samples. If needed, then these conditions could be applied to more strongly stabilized LNPs.

Due to the limited average pore size of the employed column, characterization of large mRNA may not be optimal and the suitability of the assay on a wider pore column ought to be established.^22^ The described methods achieve their maximum potential for multiple payload LNPs, however, they can also be applied to any nucleic acid loaded LNPs. We evaluated quantification of siRNA loaded LNPs achieving accurate quantification of this relatively small siRNA (20 bp) payload (Figure S15).

From a practical point of view, this method likely requires a dedicated SEC column since removal of the SDS detergent may not be entirely feasible and residual quantities of SDS are likely to interfere with other analyses. Fortuitously, we did not encounter problems in washing the LC system, which could be cleaned according to standard protocols (80% isopropanol wash) and used for other purposes with the expected performance.

## Conclusions

Efficient LC-based assays hold great potential to improve the characterization of new modality drug products. In this study, we explored the use of deformulating SEC and denaturing IP-RP methods for the quantification of multiple payload LNPs. With simple protocols, we were able to disrupt the LNP and separate the encapsulated drug substances using their differences in size (SEC) and length (IP-RP). This allowed the development of precise and fast quantification assays that can be easily applied to routine QC testing. We established that SEC with a highly deformulating mobile phase composed of organic solvent and detergent was suitable to disrupt LNPs without any need for additional sample preparation steps. Alternatively, detergent dilution of the sample and organic solvent elution in IP-RP led to the equivalent results. Both methods inform on specific impurities of the nucleic acids, such as aggregates (SEC) and lipid adducts (IP-RP). It is hoped that advances in these types of high throughput and robust methods will help reduce the time and cost burdens of developing new gene therapies.

## Supporting Information Available

Additional figures, tables, experimental details and methods, including IP-RP gradient details (PDF)
