# Supplementary material for: High-Throughput Quantification and Characterization of Dual Payload mRNA/LNP Cargo via Deformulating Size Exclusion and Ion Pairing Reversed Phase Assays
Source: Anal Chem. 2025 Jan 30;97(5):3091–8. doi: 10.1021/acs.analchem.4c06296 (PMC11822733; doi:10.1021/acs.analchem.4c06296)
Supplement: Supplementary file 1 — ac4c06296_si_001.pdf [file ac4c06296_si_001.pdf]

Supporting Information

High-Throughput Quantification and Characterization of Dual Payload mRNA/LNP Cargo via Deformulating Size Exclusion and Ion Pairing Reversed Phase Assays

Mateusz Imiołek[a*‡], Razvan Cojocaru[b‡], Szabolcs Fekete[a], Jon Le Huray[b], Matthew Lauber[c]

a - Waters Corporation, Rue Michel Servet 1 Geneva, 1211, Switzerland
b - Acuitas Therapeutics, 6190 Agronomy Rd. Suite 405, Vancouver, BC, V6T 1Z3, Canada
c - Waters Corporation, 34 Maple St. Milford, MA 01757, USA
*Corresponding author: mateusz_imiolek@waters.com, ‡These authors contributed equally.

Table of Content

Figure S1 2
Table S1 3
Figure S2 4
Figure S3 5
Figure S4 6
Figure S5 7
Table S2 8
Figure S6 9
Figure S7 10
Table S3 11
Figure S8 12
Figure S9 13
Figure S10 14
Figure S11 15
Figure S12 16
Figure S13 17
Figure S14 18
Figure S15 19

Figure S1

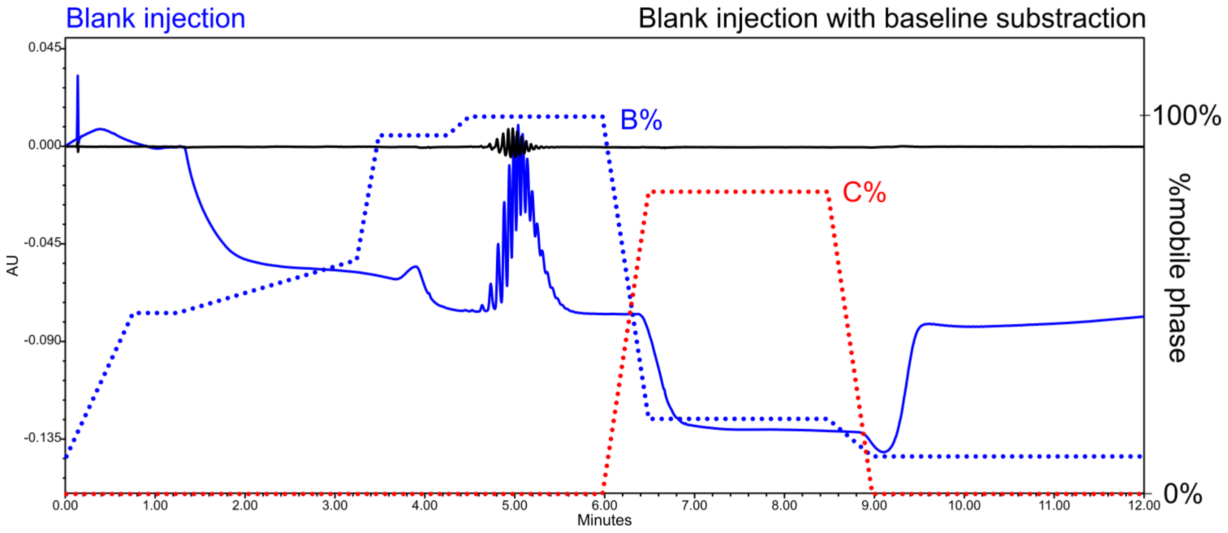

Figure S1. Overlay of IP-RP chromatograms of blank injection (blue) and blank corrected injection (black) with initial acetonitrile gradient (%B, 0 - 6 min) and subsequent isopropanol wash (%C, 6 - 8.5 min).

Table S1

Table S1. Details of the optimized multigradient elution providing satisfactory separation between sgRNA and larger mRNAs including isopropanol wash to elute residual lipids ensuring robustness of the method. In the protocol gradient 50 mM DBAA and 100 mM TEAA, pH 10 as mobile phase A, 50 mM DBAA and 100 mM TEAA in 50% MeCN as mobile phase B and 100% isopropanol as mobile phase C.

**Time**	**%A**	**%B**	**%C**
Initial	90.0	10.0	0.0
0.75	52.0	48.0	0.0
1.25	52.0	48.0	0.0
3.25	38.0	62.0	0.0
3.50	5.0	95.0	0.0
4.25	5.0	95.0	0.0
4.50	0.0	100.0	0.0
6.00	0.0	100.0	0.0
6.50	0.0	20.0	80.0
8.50	0.0	20.0	80.0
9.00	90.0	10.0	0.0
12.00	90.0	10.0	0.0

Figure S2

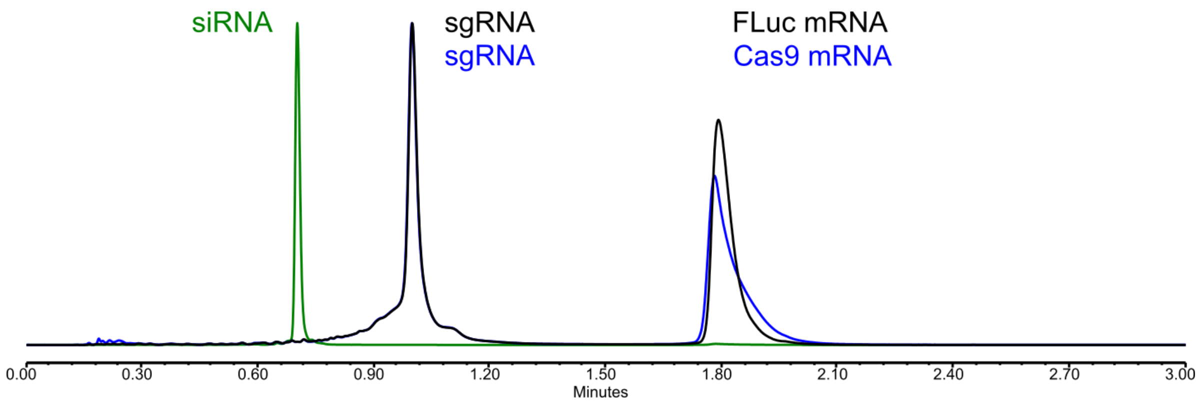

Figure S2. Overlay of normalized IP-RP chromatograms for different RNAs separated with an acetonitrile gradient and detected at 260 nm. siRNA (0.7 min, green, 20 bp), and 1:1 mixture of sgRNA (1 min, 100 nt) and Fluc mRNA (1.8 min, black, 1929 nt), or sgRNA and Cas9 mRNA (1.8 min, blue, 4521 nt). Samples were prepared in water at 0.1 mg/mL.

Figure S3

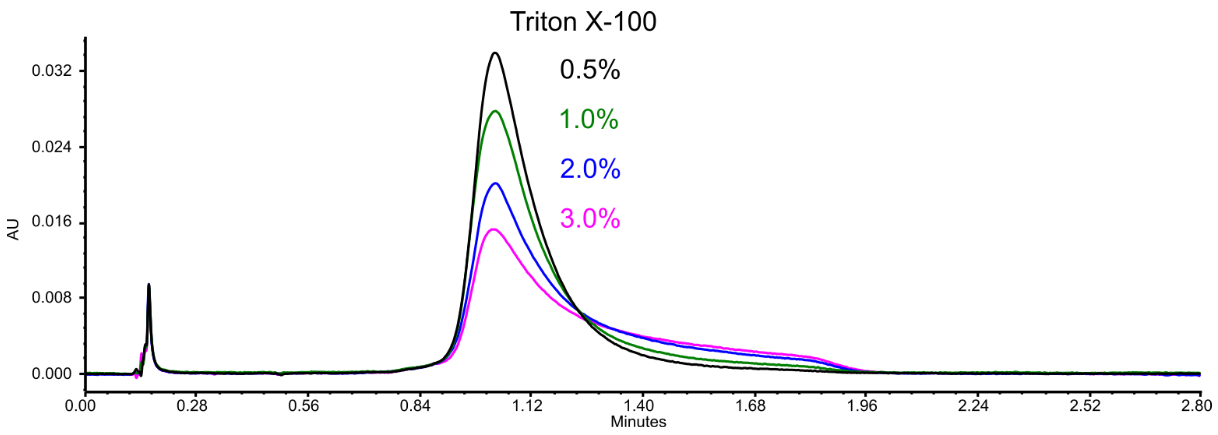

Figure S3. IP-RP separation of unencapsulated Cas9 mRNA titrated with an increasing amount of Triton[TM] X-100 Surfactant. Higher amount of detergent results in wider and more tailed RNA peaks, black (0.5%), green (1.0%), blue (2.0%), magenta (3.0%).

Figure S4

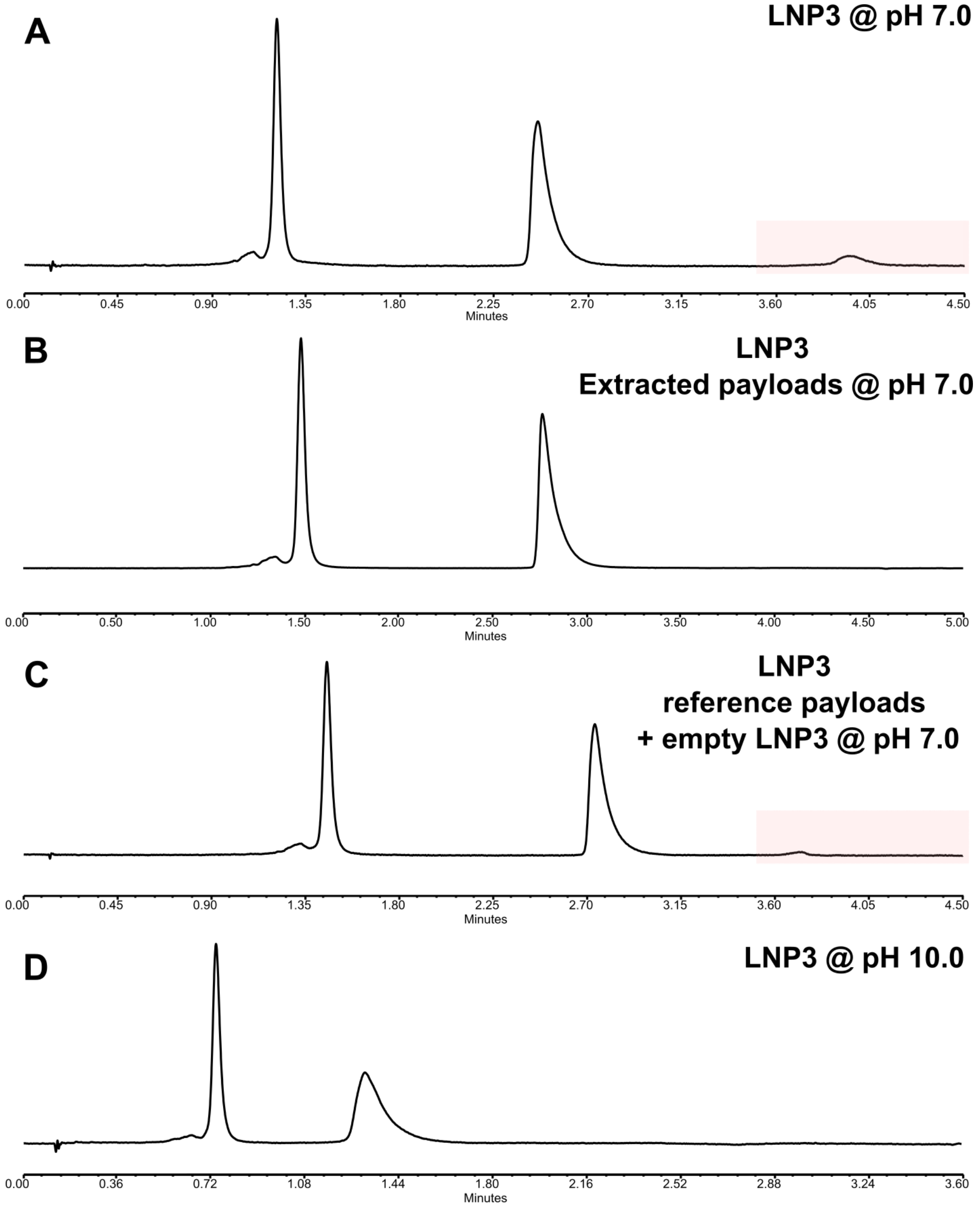

Figure S4. IP-RP separations showing late eluting peaks (red highlight) observed in deformulated samples and not present in extracted RNA. (A) deformulated LNP3, (B) extracted RNA from LNP3, (C) unencapsulated RNA spiked with empty LNP3, (D) deformulated LNP3 with pH 10 mobile phase A and B. RNA retention times vary as different gradients were used for the shown separations (while ensuring complete elution). Alcohol mediated precipitation was performed to obtain reference mRNA material from LNP3 and LNP1. Briefly, 50 µL of the LNP sample was diluted with 1 mL of 60 mM NH_4OAc in 100% IPA, which results in disruption of the particle, solubilization of the lipids, and causes encapsulated mRNA to precipitate. The samples were spun down (14,000 xg at 4 oC, 15 min), the supernatant removed, and the pellet washed with an additional 1 mL of IPA. The samples were spun down again, the supernatant removed, and the remaining pellet dried in a N_2 stream (5 min) and dissolved in 50 µL of water.

Figure S5

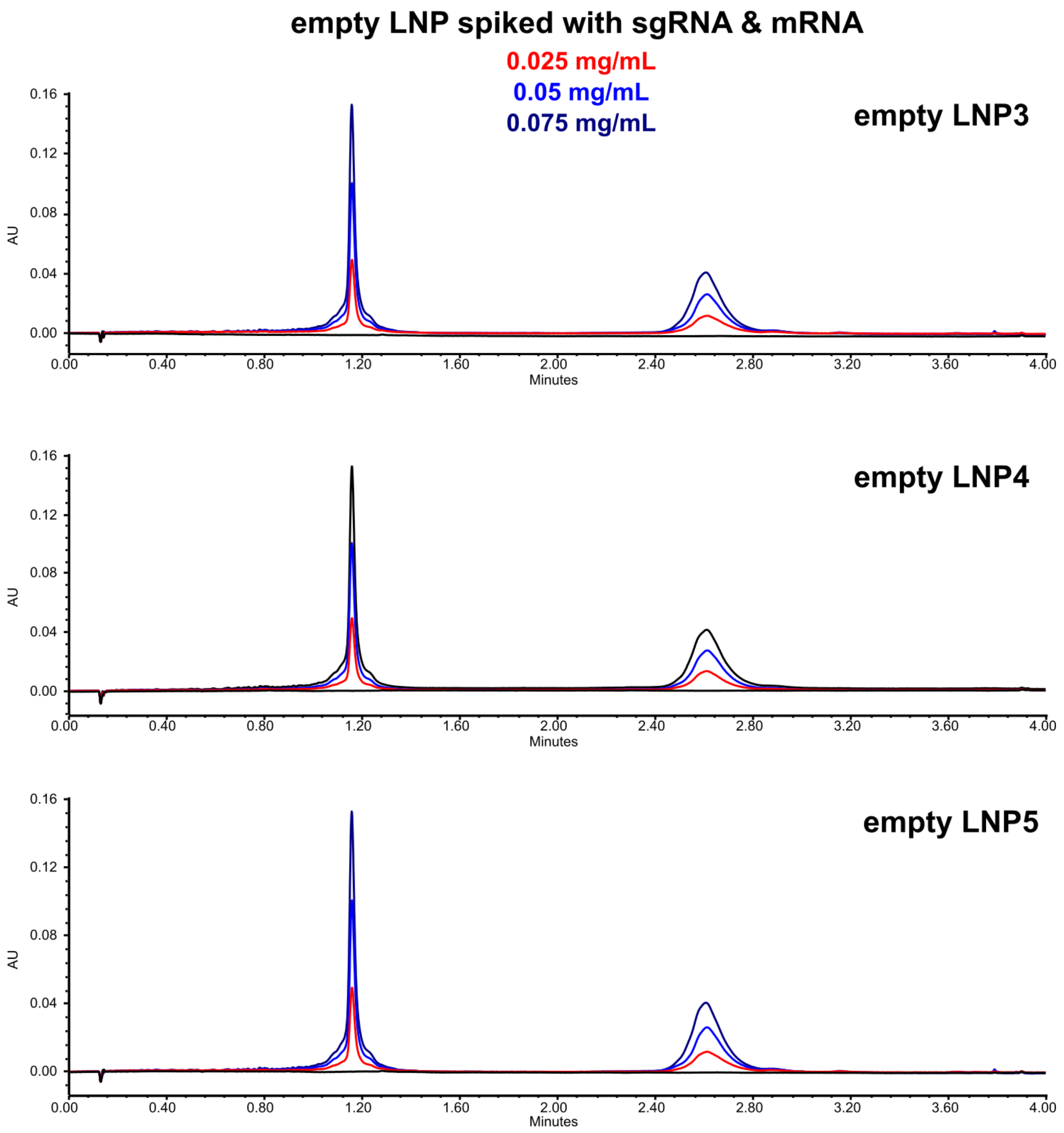

Figure S5. IP-RP chromatograms of spiking experiments for empty LNP3 (top), empty LNP4 (mid), empty LNP5 (bottom) with 0, 0.025, 0.05 and 0.075 mg/mL 1:1 mixtures of sgRNA and Fluc mRNA.

Table S2

Table S2. RNA spike recovery from empty LNPs demonstrating the accuracy of the IP RP method.

**Sample**	**RNA** [mg/mL]	**Recovery gRNA %**	**Recovery mRNA %**
LNP3 (Empty)	0.025	97.3	105.9
	0.050	100.7	103.8
	0.075	101.2	106.3
LNP4 (Empty)	0.025	92.4	104.0
	0.050	97.3	104.5
	0.075	98.8	103.9
LNP5 (Empty)	0.025	99.9	111.9
	0.05	100.4	108.9
	0.075	100.8	107.0

Figure S6

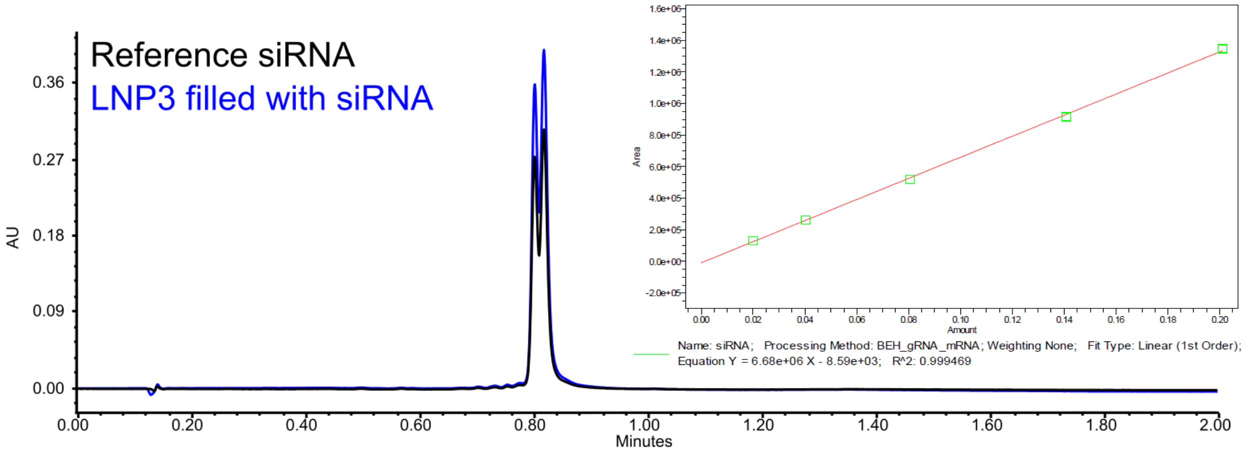

Figure S6. Deformulating IP-RP applied to siRNA reference sample (black trace) and LNP3 filled with siRNA (blue trace). Calibration curve showing linear peak area response to increasing amount of injected siRNA reference. Quantification of the LNP payload revealed amount of RNA in agreement (1.03 mg/mL) with this obtained via total RNA method (1.03 mg/mL).

Figure S7

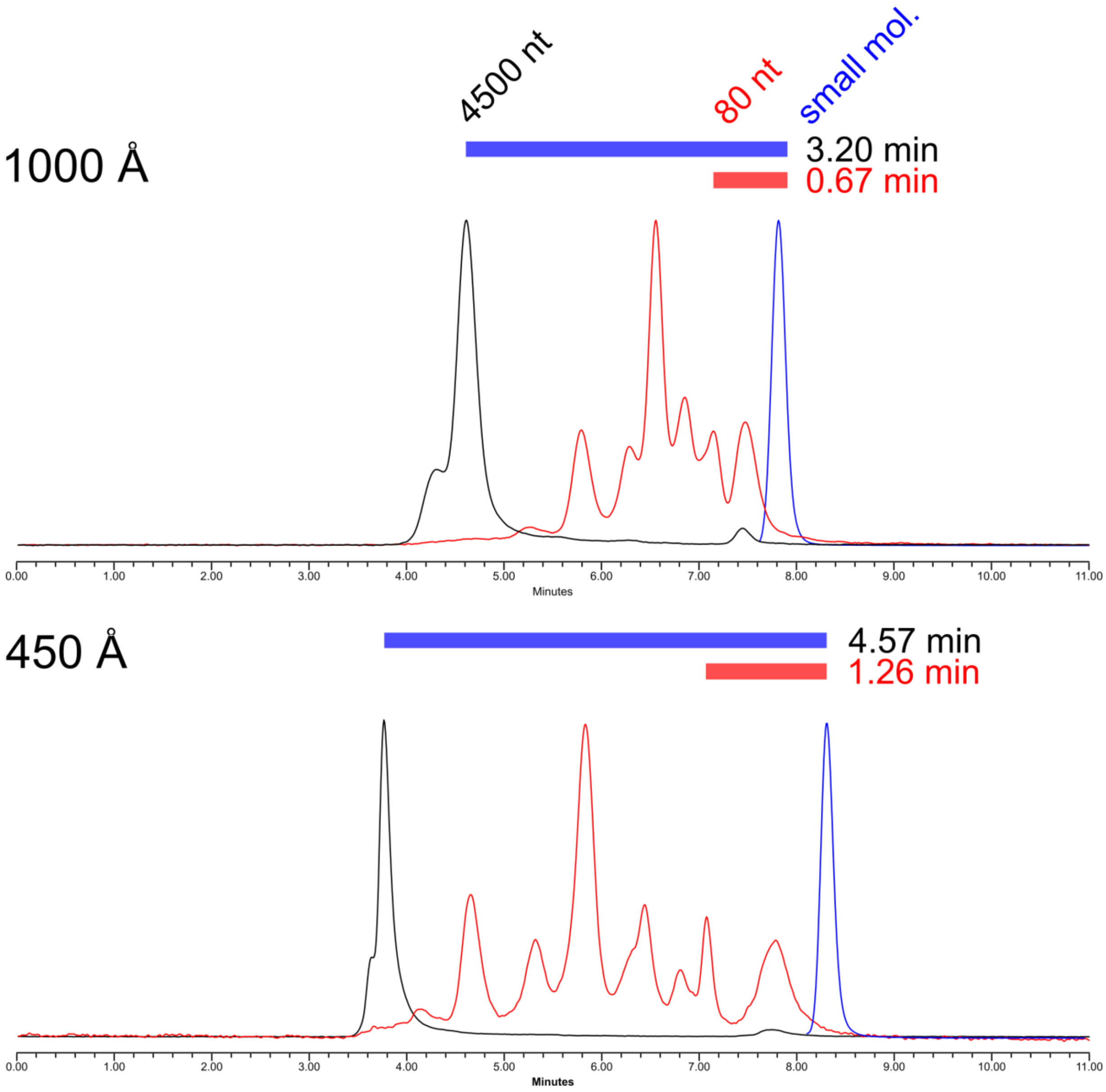

Figure S7. Overlay of normalized SEC chromatograms for different RNAs: Cas9 mRNA (black 4521 nt) low range RNA ladder (50, 80, 150, 300, 500, 1000 nt, red, please note 450 Å column allows distinguishing dimers of these species) and uracil (small molecules, blue) as acquired on GTx Resolve SEC 1000 Å (upper) and 450 Å (lower) Columns. The samples were analyzed under identical conditions and using the same column format: 100 – 500 ng, 4.6 x 150 mm, 0.25 mL/min flow rate of 1X PBS at 25 °C with detection at 260 nm.

Table S3

Table S3. SEC elution times and absorbance ratios at 260 and 230 nm for Covid vaccines LNPs, their extracted mRNA payloads and model Cas9 mRNA from native SEC analyses.

**Sample**	**Elution time (min)**	**$A_{260/230}$ ratio**
LNP1	3.908	0.93
LNP1 payload	4.033	1.99
LNP2	3.890	0.84
LNP2 payload	3.947	2.08
Cas9 mRNA	3.871	2.14

Figure S8

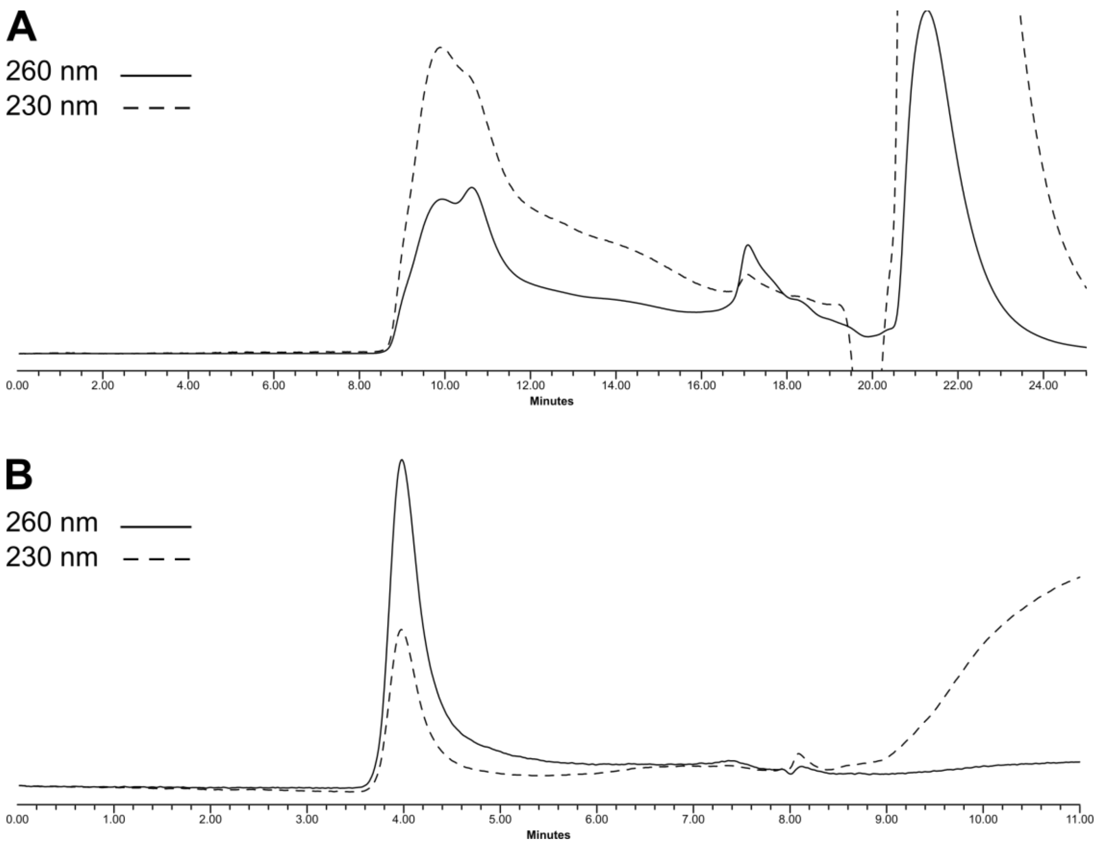

Figure S8. SEC chromatograms at 260 and 230 nm (continuous and dashed line, respectively) for A) LNP1 samples diluted with 1% Triton X-100 Surfactant (0.1 mL/min flow rate) B) LNP1 sample diluted with 1% SDS (0.25 mL/min flow rate), both analyzed under native conditions showing tailing of the main peak as well as large peak of the detergent.

Figure S9

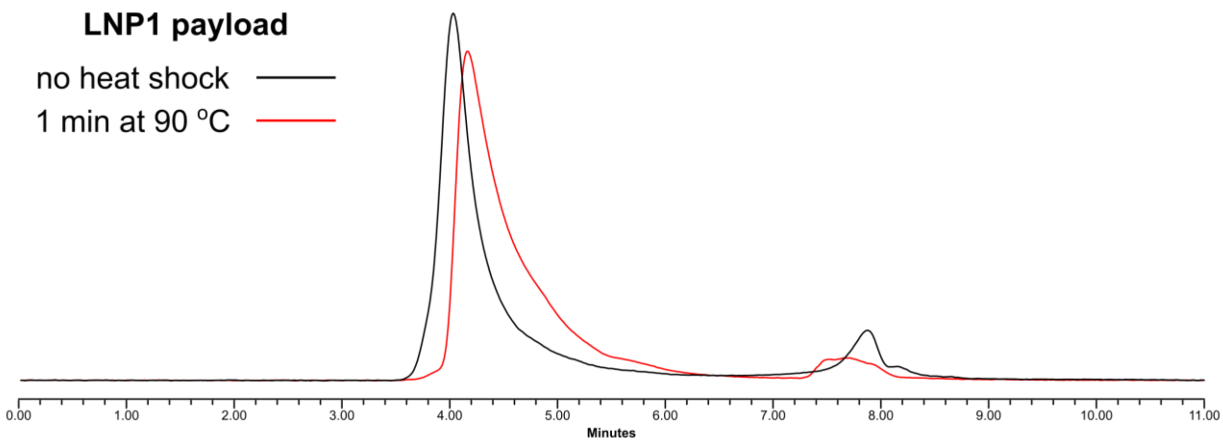

Figure S9. SEC chromatogram of extracted LNP1 mRNA payload analyzed under native conditions without (black) and with short heat shock denaturation (1 min at 90 °C).

Figure S10

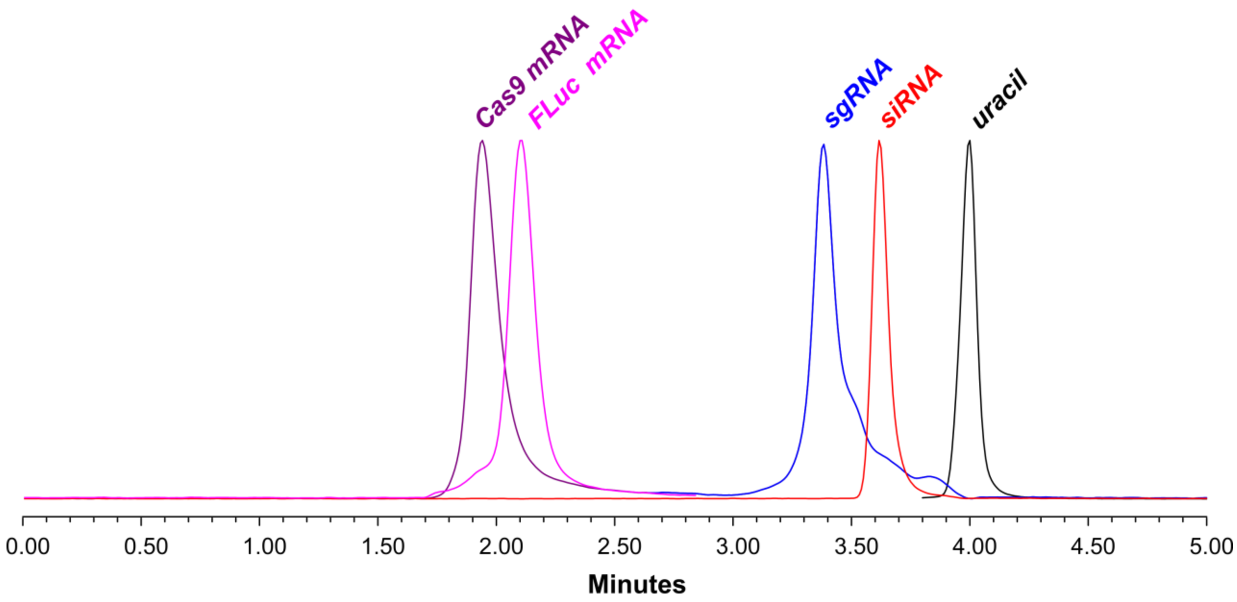

Figure S10. Overlay of SEC chromatograms showing separation of different size RNA species (Cas9 mRNA – 4521 nt, FLuc mRNA – 1929 nt, sgRNA – 100 nt, siRNA – 20 bp) of interest and small molecule (uracil) using high flow rate (0.5 mL/min) deformulating conditions (100 ng, 1X PBS, 20% IPA, 0.2% SDS at 40 ^{o}C).

Figure S11

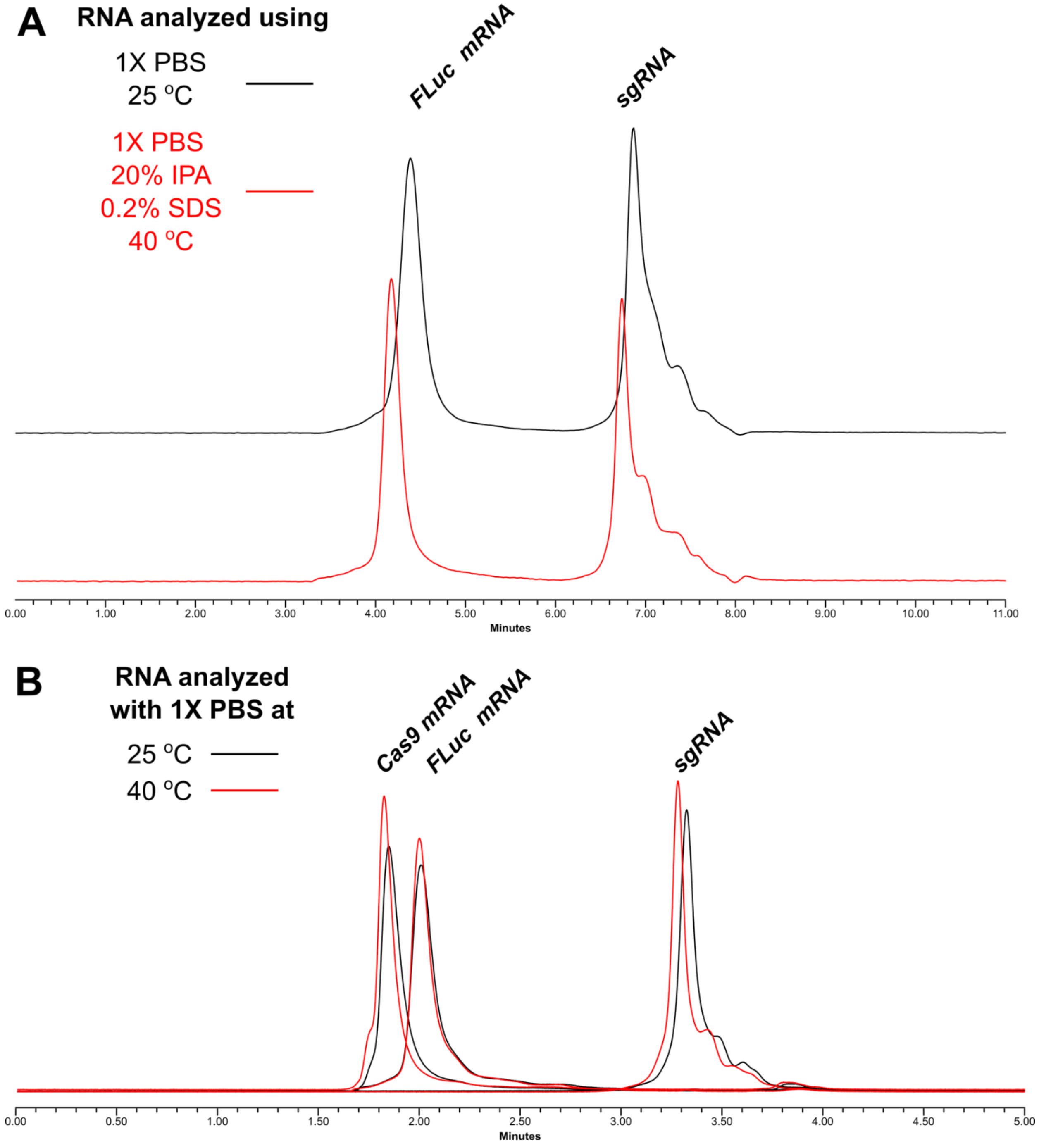

Figure S11. Analysis of impact of separation conditions on profile of RNA species of interest. A) SEC separations of FLuc mRNA and sgRNA under native (black) and deformulating (red) conditions B) Overlay of SEC chromatograms acquired under native conditions at 25 °C (black) and 40 °C demonstrating impact of temperature on separation of nucleic acids (100 ng).

Figure S12

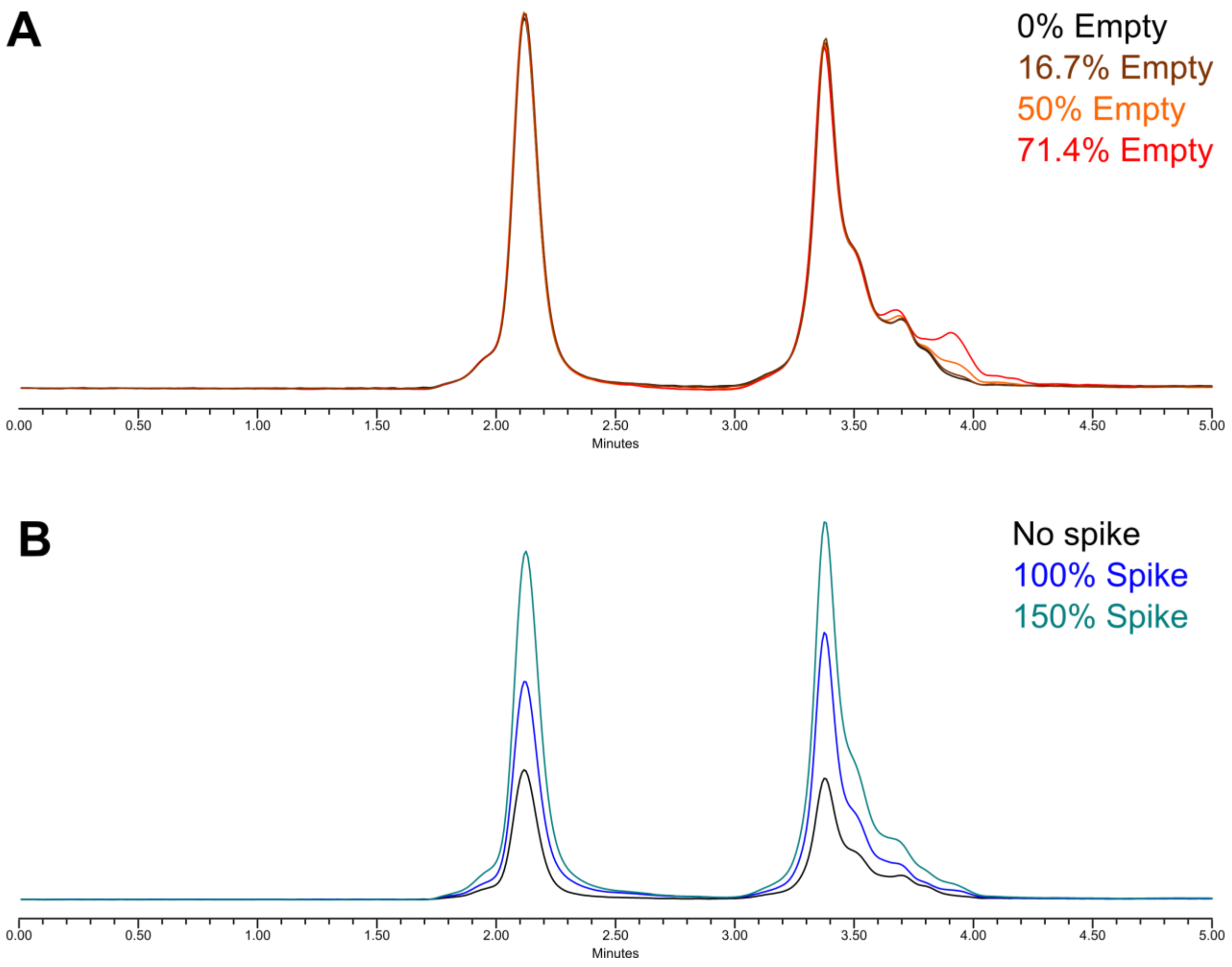

Figure S12. SEC chromatograms of spiking experiments for FLuc mRNA/sgRNA filled LNP3 (100 ng) A) co-injected with increasing amount of empty LNP3 (20, 100, 250 ng) B) co-injected with reference nucleic acids at 50 ng and 100 ng each.

Figure S13

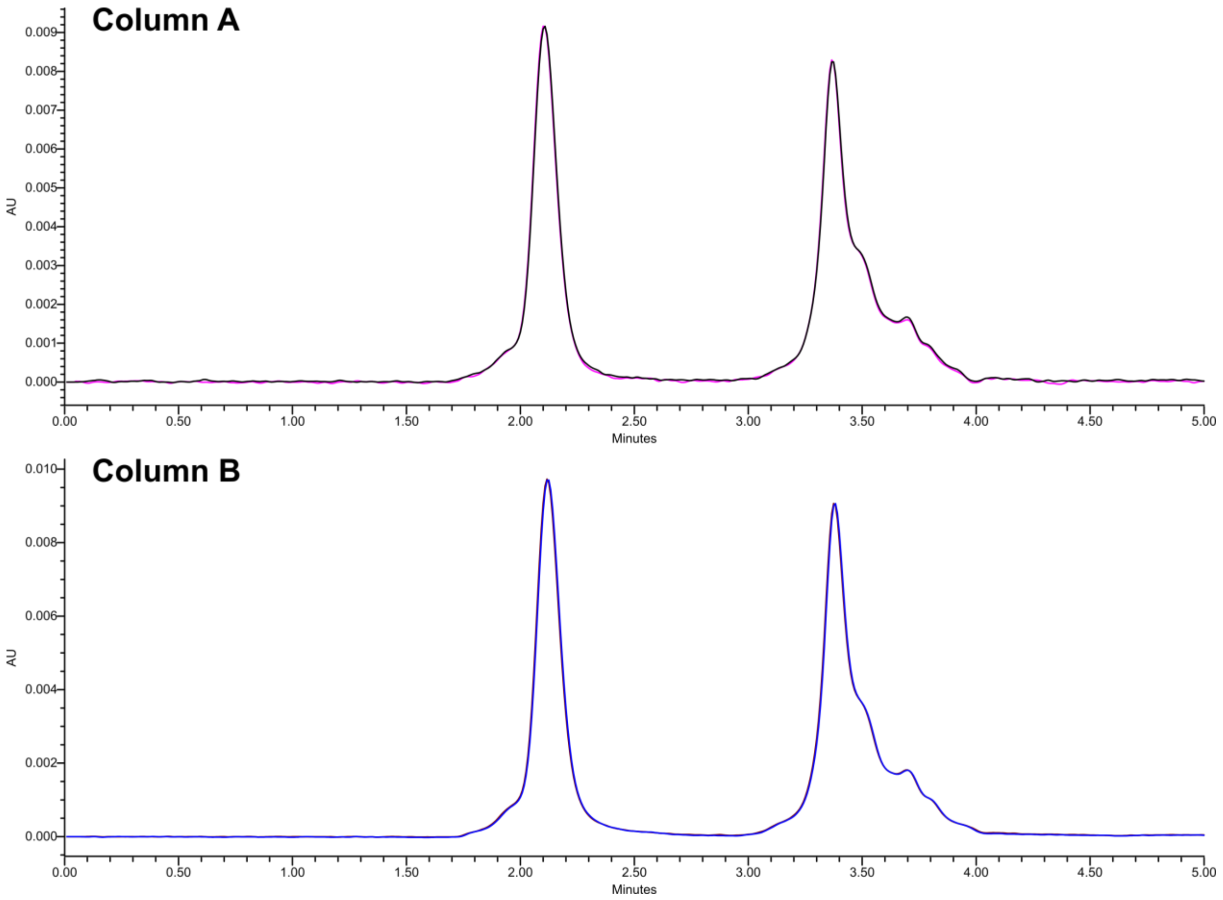

Figure S13. Overlay of triplicates SEC chromatograms acquired for the same LNP3 sample (100 ng) filled with FLuc mRNA and sgRNA injected on two SEC Columns from different batches.

Figure S14

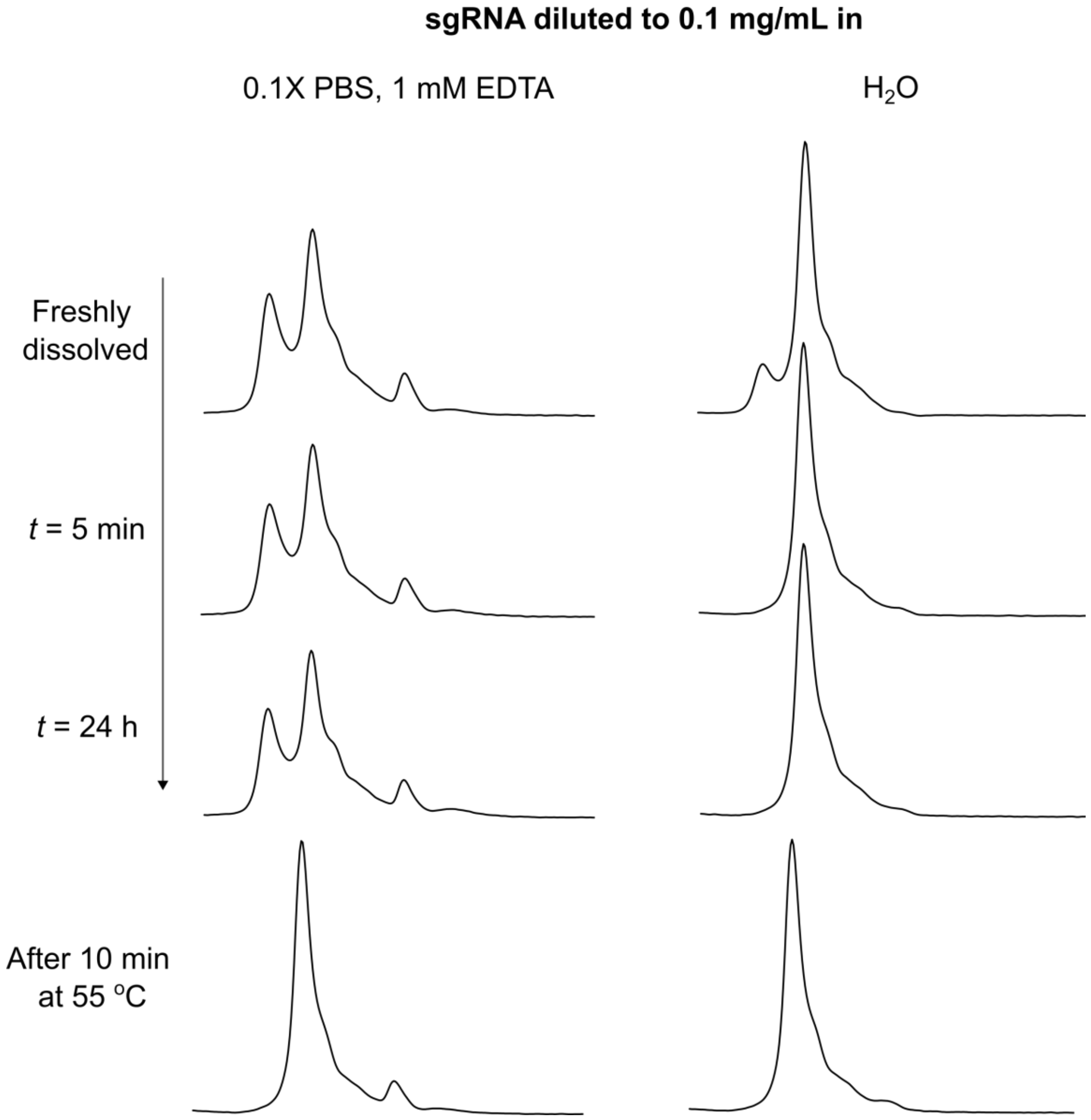

Figure S14. Time course experiment showing SEC chromatograms of sgRNA reference prepared in two different ways by 10x dilution from original formulation 1 mg/mL in 5 mM Na-citrate pH 6.0 with nuclease free waters or 0.1X PBS, 1 mM EDTA buffer. A small additional peak for sample diluted in buffer was elucidated to originate from EDTA.

Figure S15

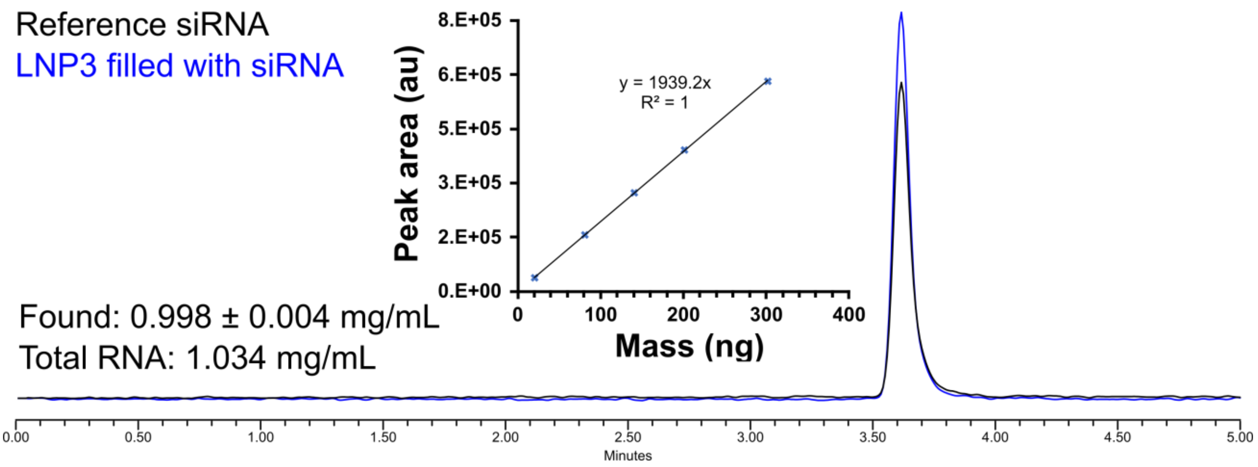

Figure S15. Deformulating SEC applied to siRNA reference sample (black trace) and LNP3 filled with siRNA (blue trace). Calibration curve showing linear peak area response to increasing amount of injected siRNA reference. Quantification of the LNP payload revealed amount of RNA in agreement with this obtained via total RNA method.
